# A prospective population-based study of maternal, fetal, and neonatal outcomes in the setting of prolonged labor, obstructed labor and failure to progress in low- and middle-income countries

**DOI:** 10.1186/1742-4755-12-S2-S9

**Published:** 2015-06-08

**Authors:** Margo S Harrison, Sumera Ali, Omrana Pasha, Sarah Saleem, Fernando Althabe, Mabel Berrueta, Agustina Mazzoni, Elwyn Chomba, Waldemar A Carlo, Ana Garces, Nancy F Krebs, K Michael Hambidge, Shivaprasad S Goudar, SM Dhaded, Bhala Kodkany, Richard J Derman, Archana Patel, Patricia L Hibberd, Fabian Esamai, Edward A Liechty, Janet L Moore, Marion Koso-Thomas, Elizabeth M McClure, Robert L Goldenberg

**Affiliations:** 1 Department of Obstetrics/Gynecology, Columbia University, New York, NY, USA; 2 Department of Community Health Sciences, Aga Khan University, Karachi, Pakistan; 3 Institute for Clinical Effectiveness and Health Policy, Buenos Aires, Argentina; 4 Tulane School of Public Health and Tropical Medicine, New Orleans, LA, USA; 5 University Teaching Hospital, University of Zambia, Lusaka, Zambia; 6 University of Alabama at Birmingham, Birmingham, AL, USA; 7 Fundación para la Alimentación y Nutrición de Centro América y Panamá (FANCAP), Guatemala City, Guatemala; 8 University of Colorado School of Medicine, Denver, CO, USA; 9 KLE University’s Jawaharlal Nehru Medical College, Belgaum, India; 10 Christiana Care Health Services, Newark, DE, USA; 11 Indira Gandhi Government Medical College and Lata Medical Research Foundation, Nagpur, India; 12 Massachusetts General Hospital for Children, Boston, MA, USA; 13 Moi University School of Medicine, Eldoret, Kenya; 14 Indiana University School of Medicine, Indianapolis, IN, USA; 15 RTI International, Durham, NC, USA; 16 Eunice Kennedy Shriver National Institute of Child Health and Human Development, Rockville, MD, USA

**Keywords:** obstructed labor, maternal mortality, maternal morbidity, neonatal mortality, neonatal morbidity, stillbirth, sub-Saharan Africa

## Abstract

**Background:**

This population-based study sought to quantify maternal, fetal, and neonatal morbidity and mortality in low- and middle-income countries associated with obstructed labor, prolonged labor and failure to progress (OL/PL/FTP).

**Methods:**

A prospective, population-based observational study of pregnancy outcomes was performed at seven sites in Argentina, Guatemala, India (2 sites, Belgaum and Nagpur), Kenya, Pakistan and Zambia. Women were enrolled in pregnancy and delivery and 6-week follow-up obtained to evaluate rates of OL/PL/FTP and outcomes resulting from OL/PL/FTP, including: maternal and delivery characteristics, maternal and neonatal morbidity and mortality and stillbirth.

**Results:**

Between 2010 and 2013, 266,723 of 267,270 records (99.8%) included data on OL/PL/FTP with an overall rate of 110.4/1000 deliveries that ranged from 41.6 in Zambia to 200.1 in Pakistan. OL/PL/FTP was more common in women aged <20, nulliparous women, more educated women, women with infants >3500g, and women with a BMI >25 (RR 1.4, 95% CI 1.3 – 1.5), with the suggestion of OL/PL/FTP being less common in preterm deliveries. Protective characteristics included parity of ≥3, having an infant <1500g, and having a BMI <18. Women with OL/PL/FTP were more likely to die within 42 days (RR 1.9, 95% CI 1.4 – 2.4), be infected (RR 1.8, 95% CI 1.5 – 2.2), and have hemorrhage antepartum (RR 2.8, 95% CI 2.1 – 3.7) or postpartum (RR 2.4, 95% CI 1.8 – 3.3). They were also more likely to have a stillbirth (RR 1.6, 95% CI 1.3 – 1.9), a neonatal demise at < 28 days (RR 1.9, 95% CI 1.6 – 2.1), or a neonatal infection (RR 1.2, 95% CI 1.1 – 1.3). As compared to operative vaginal delivery and cesarean section (CS), women experiencing OL/PL/FTP who gave birth vaginally were more likely to become infected, to have an infected neonate, to hemorrhage in the antepartum and postpartum period, and to die, have a stillbirth, or have a neonatal demise. Women with OL/PL/FTP were far more likely to deliver in a facility and be attended by a physician or other skilled provider than women without this diagnosis.

**Conclusions:**

Women with OL/PL/FTP in the communities studied were more likely to be primiparous, younger than age 20, overweight, and of higher education, with an infant with birthweight of >3500g. Women with this diagnosis were more likely to experience a maternal, fetal, or neonatal death, antepartum and postpartum hemorrhage, and maternal and neonatal infection. They were also more likely to deliver in a facility with a skilled provider. CS may decrease the risk of poor outcomes (as in the case of antepartum hemorrhage), but unassisted vaginal delivery exacerbates all of the maternal, fetal, and neonatal outcomes evaluated in the setting of OL/PL/FTP.

## Background

Obstructed labor (OL) is a common cause of maternal mortality, accounting for approximately 6% of maternal deaths worldwide and substantial long-term maternal morbidity [1]. Maternal mortality from OL is caused by ruptured uterus, postpartum hemorrhage, and puerperal sepsis, while maternal morbidity includes secondary infertility, vaginal scarring and stenosis, severe anemia, musculoskeletal injury, urinary incontinence and obstetric fistula [2,3]. OL also has implications for the fetus or neonate — it frequently results in asphyxia that can result in stillbirth, neonatal demise, cerebral palsy, and developmental disabilities [4].

According to the World Health Organization, labor is obstructed when the presenting part of the fetus cannot progress into the birth canal despite strong uterine contractions [1]. The etiology is often cephalo-pelvic disproportion (CPD), which is defined as a mismatch between the size of the fetal presenting part and the mother’s pelvis [2]. Often, in developing countries, CPD is due to stunted growth of the maternal pelvic bones from malnutrition, early childbearing before the growth of the pelvis is complete, or abnormalities of the shape of the pelvis due to rickets or osteomalacia [5].

While there is literature on maternal mortality resulting from OL, the complexity of isolating OL as the cause of any individual maternal, fetal, or neonatal death makes data collection and analysis difficult and often of poor quality. After performing a comprehensive literature review for stillbirth and neonatal outcomes related to OL, only two small, single institution studies were found that evaluated perinatal outcomes in pregnancies complicated by OL [6,7]. Thus, we sought to undertake a review of a large, prospective study on pregnancy, the Global Network’s Maternal Newborn Health Registry (MNHR). Reviewing the experience illustrated by the MNHR data will shed light on both maternal and perinatal morbidity and mortality associated with to OL in low- and middle-income countries.

## Methods

This data analysis was conducted on information from a prospective population-based observational study conducted in 106 communities at six sites in five low-income countries on births from January 1, 2010 through December 31, 2013 (Chimaltenango, Guatemala; Nagpur, India; Belgaum District, India; western Kenya; Thatta District, Pakistan; and Lusaka, Zambia) and at one site in a middle-income country (Corrientes, Argentina). These seven sites were selected by the *Eunice Kennedy Shriver* National Institute of Child Health and Human Development in the United States of America (NICHD), a governmental organization that supports the Global Network for Women’s and Children’s Health Research (GN), which is a network of research institutions in the aforementioned sites that enrolls women during pregnancy and collects data through 6-weeks postpartum to assess pregnancy outcomes.

The prospective community-based registry, called the Maternal and Newborn Health Registry (MNHR), includes outcomes from rural or semi-urban geographical areas served by government health services. Each site includes between six and 24 distinct communities. The methods of the MNHR have been published [8]. In general, each community represents the catchment area of a primary healthcare center, and about 300 to 500 births take place annually in each locale. Beginning in 2009 and 2010, the study investigators at each site initiated an ongoing, prospective maternal and newborn health registry of pregnant women for each community. The objective is to enroll pregnant women by 20 weeks’ gestation and to obtain data on pregnancy outcomes for all deliveries that take place in the community. Each community employs a registry administrator who identifies and tracks pregnancies and their outcomes in coordination with community elders, birth attendants, and other health care workers.

The primary purpose of the MNHR is to quantify and analyze trends in pregnancy outcomes in defined low-resource geographic areas over time in order to provide population-based statistics on pregnancy outcomes, including stillbirths, neonatal, and maternal mortality. This analysis utilizes the MNHR to determine maternal and fetal outcomes in the setting of dysfunctional labor and to compare these outcomes to a reference population, also from the registry, that did not experience this labor complication. In these settings it is difficult to define dysfunctional labor because it is nearly impossible to distinguish clinically between obstructed labor, prolonged labor, and/or failure to progress in labor, so for the purposes of data collection, these outcomes were combined into a single overall outcome called obstructed labor/prolonged labor/failure to progress (OL/PL/FTP).

The definition of OL/PL/FTP in the MNHR is, “a situation when the descent of the presenting part is arrested during labor due to an insurmountable barrier. This occurs in spite of strong uterine contractions and further progress cannot be made without assistance. Obstruction usually occurs at the brim but it may occur in the cavity or at the outlet of the pelvis”. This definition is adapted from the World Health Organization’s definition, noted in the introduction. All sites involved in this analysis used the same definition for OL/PL/FTP.

Other co-variates were defined in accordance with the WHO definitions, described elsewhere [9]. Specifically, body mass index (BMI), in kg/m^2^, was calculated based upon weight and maternal height taken at the antenatal care visit (the Kenya site did not obtain BMI measurements and were omitted from those analyses with BMI). Gestational age (GA) at delivery was determined as term (≥37 weeks gestation) or preterm (<37 weeks) for all deliveries, based on last menstrual period (LMP) or ultrasound, when available, and finally, birth weight was the weight of the live birth or stillbirth taken at the delivery visit.

Data were collected and entered into research computers at each study site and transmitted through secure methods to a central data coordinating center (RTI International). All analyses were done with SAS version 9.3 (SAS Institute, Cary, NC, USA). Analyses included descriptive statistics. Relative risks were computed using generalized estimating equations, accounting for study clusters. In addition, because the findings related to education were unexpected, an additional regression analysis was run to better understand the relationship between OL/PL/FTP and maternal education.

The appropriate institutional review boards/ethics research committees of the participating institutions and the ministries of health of the respective countries approved the MNHR. Prior to initiation of the study, approval was sought from the participating communities through sensitization meetings. Individual informed consent for study participation is requested from each study participant. Monetary reimbursements are not provided to study participants nor to the communities participating in the study. A Data Monitoring Committee, appointed by the NICHD, oversees and reviews the study at annual meetings.

## Results

Between 2010 and 2013, 266,723 of 267,270 records (99.8%) included data on whether or not the woman experienced OL/PL/FTP. For the women with information on OL/PL/FTP, 62% of deliveries were in Southeast Asia, 23% at the African sites, and 15% of the deliveries took place in Latin American sites. In the population studied, the vaginal delivery rate was 86.2%, the operative vaginal delivery rate was 1.6%, and the cesarean section rate was 12.2%. In the setting of OL/PL/FTP, the rate of operative vaginal delivery increased from 0.9% to 6.6%, and cesarean section rate increased from 7% to 53%, which represented seven and eight-fold increases over no OL/PL/FTP, respectively.

**Figure 1:**
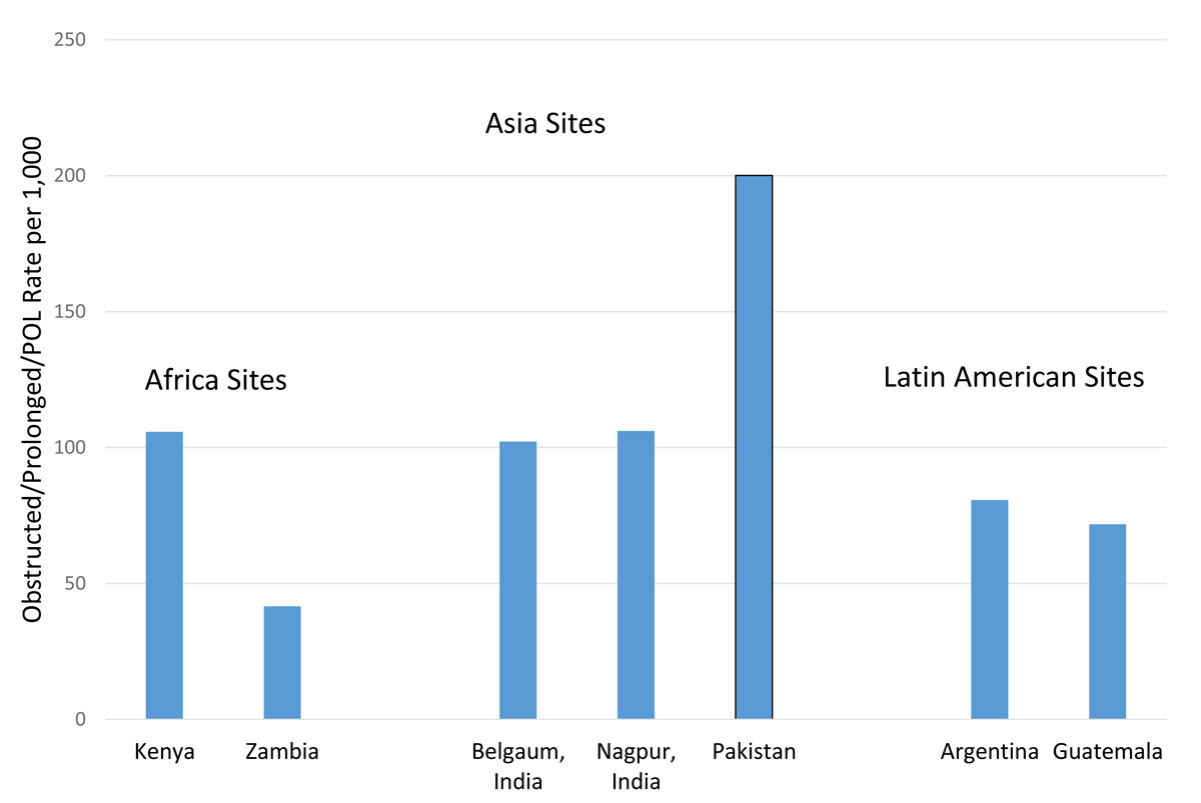
Rates of OL/PL/FTP per 1000 deliveries by Site, 2010-2013

Figure 1 graphically represents the OL/PL/FTP rate in each community, with an overall rate of 110.4/1000 deliveries in the whole cohort. The rates of OL/PL/FTP ranged from 41.6/1000 births in Zambia to 200.1/1000 in Pakistan.

Table 1 illustrates the demographic characteristics of the women involved in the study. In the subpopulation of women experiencing OL/PL/FTP as well as in the general population, the age distribution was similar with about 84% aged between 20 – 35, about 12% younger than 20, and the remainder being over 35. The youngest women (age <20 years) had a 30% (RR 1.3, 95% CI 1.2 – 1.3) increased risk of experiencing OL/PL/FTP as compared to the 20 – 35 age group, which encompassed the majority of women. Compared to women who had one or two prior deliveries, women in their first pregnancy were 80% (RR 1.8, 95% CI 1.7 – 2.0) more likely to experience OL/PL/FTP; conversely, women who had already had three or more prior deliveries were 20% (RR 0.8, 95% CI 0.7 – 0.9) less likely to experience obstruction. Interestingly, unlike parity, more education was associated with increased risk of a woman experiencing OL/PL/FTP. Compared to the referent group of women with a primary school education, the risk of having OL/PL/FTP was almost two fold higher in the most highly educated women—those with a university level education (RR 1.8, 95% CI 1.7 – 1.9). Women with no formal education had a reduced risk of OL/PL/FTP (RR 0.7, 95% CI 0.7 – 0.8). As this was an unexpected finding, an additional regression analysis was performed on these data, including an adjustment for age, maternal education and parity. Findings were unchanged, still showing that compared to a primary school level of education, no education was a protective characteristic in terms of OL/PL/FTP, while having a secondary level education and university level education were both associated with increased risk of OL/PL/FTP.

**Table 1:** Maternal and Delivery Characteristics of Women Experiencing OL/PL/FTP vs Normal Labor, 2010-2013

	OL/PL/FTP	No OL/PL/FTP	RR (95% CI)
Women with deliveries, N*	29,449	237,274	

Maternal age, N (%)			

< 20	3,503 (11.9)	28,713 (12.1)	1.3 (1.2, 1.3)

20-35	24,929 (84.8)	198,840 (83.9)	1.0

> 35	950 (3.2)	9,360 (4.0)	1.0 (0.9, 1.0)

Parity, N (%)			

0	14,074 (48.0)	75,629 (32.0)	1.8 (1.7, 2.0)

1-2	9,436 (32.2)	102,628 (43.4)	1.0

≥3	5,816 (19.8)	58,359 (24.7)	0.8 (0.7, 0.9)

Education, N (%)			

No formal education	8,748 (29.8)	57,912 (24.5)	0.7 (0.7, 0.8)

Primary	8,278 (28.2)	92,430 (39.1)	1.0

Secondary	8,996 (30.7)	70,354 (29.8)	1.3 (1.2, 1.3)

University+	3,311 (11.3)	15,488 (6.6)	1.8 (1.7, 1.9)

Birth weight (measured), N (%)			

< 1500g	140 (0.5)	1,852 (0.8)	0.7 (0.5, 0.8)

1500-2499g	3,194 (12.0)	23,170 (10.5)	1.0 (1.0, 1.1)

2500-3499g	20,349 (76.6)	173,974 (79.0)	1.0

> 3500g	2,894 (10.9)	21,163 (9.6)	1.2 (1.1, 1.3)

Gestational age at delivery, N (%)			

Preterm	2,589 (9.2)	22,182 (9.6)	0.9 (0.8, 1.0)

Term	25,683 (90.8)	208,047 (90.4)	1.0

			

BMI, N (%)	23,857	182,118	

< 18 kg/m^2^	4,056 (17.0)	32,459 (17.8)	0.8 (0.8, 0.9)

18-25 kg/m^2^	16,120 (67.6)	124,468 (68.3)	1.0

> 25 kg/m^2^	3,681 (15.4)	25,191 (13.8)	1.4 (1.3, 1.5)

Delivery mode, N (%)			

Vaginal	12,022 (40.8)	217,381 (91.9)	1.0

Vaginal assisted	1,933 (6.6)	2,194 (0.9)	7.1 (5.1, 9.8)

Cesarean section	15,488 (52.6)	17,061 (7.2)	10.0 (7.8, 12.8)

Birth attendant, N (%)			

Physician	20,133 (68.4)	82,183 (34.6)	18.1 (9.5, 34.3)

Nurse/Midwife/HW	6,558 (22.3)	80,998 (34.2)	5.2 (2.9, 9.4)

TBA/Family/Other	2,754 (9.4)	74,002 (31.2)	1.0

Delivery location, N (%)			

Hospital	21,491 (73.0)	100,822 (42.5)	14.5 (8.3, 25.2)

Clinic	5,376 (18.3)	62,454 (26.3)	4.9 (2.9, 8.5)

Home/Other	2,569 (8.7)	73,869 (31.1)	1.0

*numbers less than the total reflect missing data.

With respect to birthweight, which can also been seen in Table 1, OL/PL/FTP was more common in larger fetuses. Fetuses <1500g were less likely to have OL/PL/FTP (RR 0.7, 95% CI 0.5 – 0.8), and fetuses ≥ 3500g more likely to have OL/PL/FTP than women with a fetus with birthweight of 2500 – 3499g (RR 1.2, 95% CI 1.1 – 1.3). Deliveries categorized as preterm were about 10% less likely to be complicated by OL/PL/FTP than those pregnancies that were carried to term. Data on BMI, suggested that heavier women (BMI >25) were 40% more likely to experience OL/PL/FTP (RR 1.4, 95% CI 1.3 – 1.5) than women with a BMI 18 – 25 kg/m^2^; leaner women (BMI < 18 kg/m^2^) were 20% less likely to have OL/PL/FTP than the reference population (RR 0.8, 95% CI 0.8-0.9). Finally, in terms of attendants at delivery, women with OL/PL/FTP were much more likely to be delivered in the hospital or clinic than at home (RR 14.5, 95% CI 8.3 – 25.2; RR 4.9, 95% CI 2.9 – 8.5), and much more likely to be delivered by a physician or nurse/midwife/healthcare worker than by a traditional birth attendant/family member/or other provider (RR 18.1, 95% CI 9.5 – 34.3; RR 5.2, 95% CI 2.9 – 9.4).

Table 2 illustrates that across all seven sites and all outcomes related to maternal morbidity and mortality, every complication was significantly increased in women who experienced OL/PL/FTP, except for maternal mortality in Latin America. This result in Latin America is likely the result of small sample size as only one labor was complicated by a maternal death attributed to OL/PL/FTP (RR 0.4, 95% CI 0.1 – 2.1). The outcomes of interest included 42-day maternal mortality, maternal infection, and antepartum and postpartum hemorrhage. All outcomes were about twice more likely to occur in labors complicated by OL/PL/FTP than those that were not. Of particular interest in this analysis is the fact that African women experienced more morbidity and mortality than women in Asia and Latin America who also had OL/PL/FTP, with relative risks ranging from 3.4 (in the case of infection) to 9.1 for antepartum hemorrhage.

**Table 2:** Maternal Morbidity and Mortality in Women Experiencing OL/PL/FTP vs Normal Labor by Region, 2010-2013

	Africa			Asia			Latin America			Total		
	
	OL/PL/FTP	No OL/PL/FTP	RR (95% CI)	OL/PL/FTP	No OL/PL/FTP	RR (95% CI)	OL/PL/FTP	No OL/PL/FTP	RR (95% CI)	OL/PL/FTP	No OL/PL/FTP	RR (95% CI)
42 day maternal mortality, n/N (Rate/100,000 deliveries)	21/4,833 (435)	52/57,327 (91)	4.8 (2.7, 8.7)	50/21,578 (232)	214/142,967 (150)	1.5 (1.1, 2.0)	1/2,861 (35)	32/35,853 (89)	0.4 (0.1, 2.1)	72/29,272 (246)	298/236,147 (126)	1.9 (1.4, 2.4)

Maternal infection, n/N (%)	93/4,820 (1.9)	217/57,284 (0.4)	3.4 (2.0, 5.9)	295/21,429 (1.4)	1,001/141,547 (0.7)	1.5 (1.3, 1.8)	21/2,856 (0.7)	130/35,812 (0.4)	1.8 (1.1, 3.2)	409/29,105 (1.4)	1,348/234,643 (0.6)	1.8 (1.5, 2.2)

Antepartum hemorrhage^2^, n/N (%)	520/4,855 (10.7)	623/57,616 (1.1)	9.1 (5.3, 15.7)	815/21,692 (3.8)	2,187/143,579 (1.5)	1.8 (1.5, 2.1)	103/2,874 (3.6)	431/36,060 (1.2)	2.9 (2.2, 3.7)	1,438/29,421 (4.9)	3,241/237,255 (1.4)	2.8 (2.1, 3.7)

Postpartum hemorrhage^3^, n/N (%)	882/4,841 (18.2)	1,434/57,605 (2.5)	5.0 (2.8, 9.0)	689/21,692 (3.2)	1,748/143,584 (1.2)	1.5 (1.2, 1.8)	120/2,872 (4.2)	649/36,048 (1.8)	2.0 (1.4, 2.9)	1,691/29,405 (5.8)	3,831/237,237 (1.6)	2.4 (1.8, 3.3)

Similar to the results shown in Table 2, Table 3 also shows that stillbirths, neonatal mortality, and neonatal infection occurred more often in women with OL/PL/FTP than those who did not have this diagnosis, with RR of 1.6 (95% CI 1.3 – 1.9), 1.9 (95% CI 1.6 – 2.1), and 1.2 (95% CI 1.1 – 1.3), respectively. Additionally, the data again showed poorer outcomes in African women in the case of stillbirth (RR 4.8, 395% CI.7 – 6.1) and neonatal mortality (RR 3.6, 95% CI 3.0 – 4.4), but not in neonatal infection, where neonates in each location born of a labor complicated by OL/PL/FTP experienced a 20% increased risk of infection (RR 1.2, 95% CI 1.1 – 1.4).

**Table 3:** Stillbirth and Neonatal Morbidity and Mortality in Women Experiencing OL/PL/FTP vs Normal Labor by Region, 2010-2013

	Africa			Asia			Latin America			Total		
	
	OL/PL/FTP	No OL/PL/FTP	RR (95% CI)	OL/PL/FTP	No OL/PL/FTP	RR (95% CI)	OL/PL/FTP	No OL/PL/FTP	RR (95% CI)	OL/PL/FTP	No OL/PL/FTP	RR (95% CI)
Stillbirths, n/N (Rate/1,000)	340/4,860 (70.0)	909/57,615 (15.8)	4.8 (3.7, 6.1)	952/21,700 (43.9)	4,594/143,513 (32.0)	1.2 (1.0,1.4)	86/2,883 (29.8)	564/36,066 (15.6)	2.0 (1.5, 2.5)	1,378/29,443 (46.8)	6,067/237,194 (25.6)	1.6 (1.3, 1.9)

Neonatal mortality<28d, n/N (Rate/1,000)	182/4,496 (40.5)	680/56,439 (12.0)	3.6 (3.0, 4.4)	980/20,632 (47.5)	3,529/138,313 (25.5)	1.7 (1.4,1.9)	72/2,776 (25.9)	595/35,293 (16.9)	1.6 (1.2, 2.1)	1,234/27,904 (44.2)	4,804/230,045 (20.9)	1.9 (1.6, 2.1)

Infection, n/N (%)	631/4,491 (14.1)	3,496/56,429 (6.2)	1.2 (1.1, 1.4)	2,234/20,627 (10.8)	8,536/138,297 (6.2)	1.2 (1.1,1.4)	216/2,773 (7.8)	2,200/35,271 (6.2)	1.2 (1.1, 1.4)	3,081/27,891 (11.0)	14,232/229,997 (6.2)	1.2 (1.1, 1.3)

Table 4 displays the outcomes of women experiencing OL/PL/FTP by method of delivery, which include spontaneous vaginal delivery, operative vaginal delivery, and cesarean section. The analysis shows that delivery by cesarean section only improves maternal antepartum hemorrhage in the setting of OL/PL/FTP, but does not have an association with maternal mortality, maternal infection postpartum hemorrhage, the stillbirth rate, neonatal mortality, or neonatal infection. Women who were designated as having OL/PL/FTP but were eventually delivered vaginally without assistance (e.g. without the use of forceps or vacuum), were more likely to experience every single adverse outcome. Women with spontaneous vaginal births after OL/PL/FTP were about three times more likely to succumb, to have a stillbirth, and to have a neonatal death (RR 3.0, 95% CI 2.0 – 4.5; RR 3.3, 95% CI 2.8 – 3.9; RR 3.0, 95% CI 2.5 – 3.6), 60% more likely to have maternal infection (RR 1.6, 95% CI 1.3 – 2.1), almost five times more likely to experience antepartum hemorrhage (RR 4.7, 95% CI 3.4 – 6.7), about four times more likely to have a delivery complicated by postpartum hemorrhage (RR 3.9, 95% CI 2.7 – 5.6), and were 40% more likely to have a neonate with an infection (RR 1.4, 95% CI 1.2 – 1.6).

**Table 4:** Outcomes for OL/PL/FTP by Delivery Mode, 2010-2013

	OL/PL/FTP	No OL/PL/FTP	RR (95% CI)
42 day maternal mortality, n/N (Rate/100,000 women)			

Vaginal	31/11,937 (260)	181/216,366 (84)	3.0 (2.0, 4.5)

Operative Vaginal	4/1,916 (209)	9/2,170 (415)	0.5 (0.2, 1.4)

Cesarean Section	35/15,414 (227)	35/16,979 (206)	1.0 (0.6, 1.6)

Maternal infection, n/N (%)			

Vaginal	137/11,894 (1.2)	1,177/215,055 (0.5)	1.6 (1.3, 2.1)

Operative Vaginal	33/1,909 (1.7)	60/2,159 (2.8)	1.4 (0.9, 2.2)

Cesarean Section	239/15,299 (1.6)	108/16,871 (0.6)	1.1 (1.0, 1.4)

Antepartum hemorrhage, n/N (%)			

Vaginal	893/12,014 (7.4)	2,534/217,366 (1.2)	4.7 (3.4, 6.7)

Operative Vaginal	163/1,932 (8.4)	103/2,194 (4.7)	1.5 (1.1, 2.2)

Cesarean Section	380/15,470 (2.5)	521/17,057 (3.1)	0.6 (0.5, 0.7)

Postpartum hemorrhage, n/N (%)			

Vaginal	1,380/12,010 (11.5)	3,563/217,353 (1.6)	3.9 (2.7, 5.6)

Operative Vaginal	150/1,931 (7.8)	94/2,194 (4.3)	2.0 (1.5, 2.7)

Cesarean Section	161/15,459 (1.0)	153/17,052 (0.9)	1.2 (0.9, 1.4)

Stillbirths, n/N (Rate/1,000)			

Vaginal	989/12,020 (82.3)	5,099/217,377 (23.5)	3.3 (2.8, 3.9)

Operative Vaginal	134/1,931 (69.4)	144/2,194 (65.6)	1.1 (0.8, 1.5)

Cesarean Section	252/15,488 (16.3)	265/17,060 (15.5)	0.9 (0.8, 1.1)

Neonatal mortality < 28 days, n/N (Rate/1,000)			

Vaginal	731/10,951 (66.8)	4,292/211,303 (20.3)	3.0 (2.5, 3.6)

Operative Vaginal	112/1,783 (62.8)	91/2,027 (44.9)	1.4 (1.0, 2.0)

Cesarean Section	391/15,169 (25.8)	421/16,711 (25.2)	0.9 (0.7, 1.0)

Neonatal infection, n/N (%)			

Vaginal	1,618/10,942 (14.8)	13,173/211,268 (6.2)	1.4 (1.2, 1.6)

Operative Vaginal	398/1,783 (22.3)	313/2,027 (15.4)	1.1 (0.9, 1.5)

Cesarean Section	1,064/15,165 (7.0)	745/16,698 (4.5)	1.1 (1.0, 1.1)

## Discussion

This population-based study provides estimates of the rate of OL/PL/FTP in 7 sites in 6 countries in a population-based study of more than 260,000 births. Women with OL/PL/FTP were more likely to be primiparous, younger than age 20, with a BMI > 25 kg/m^2^ and of higher education, with a fetal birthweight of >3500 g. Women with this diagnosis were more likely to experience a maternal, fetal, or neonatal death, antepartum and postpartum hemorrhage, and maternal and neonatal infection. Outcomes were often worse in women experiencing OL/PL/FTP in Africa compared to the other locations.

Our literature review for maternal and perinatal outcomes related to obstructed labor found small, single institution studies that evaluate perinatal outcomes in pregnancies complicated by obstructed labor [6,7]. One study from Nigeria that evaluated 120 perinatal outcomes in the setting of OL found a 23% stillbirth rate and a 6.7% early neonatal death rate [6]. Our analysis, which assessed the outcomes of more than 29,000 labors complicated by OL/PL/FTP found a stillbirth rate of 46.8/1000 deliveries and 44.2 neonatal deaths per 1000 live births. In a study from Sudan, which reported on the outcomes of 42 women experiencing OL, the rate of sepsis (not specified as maternal or neonatal) was 7.1%, postpartum hemorrhage 11.9%, maternal death 4.8%, stillbirth 26.2%, and early neonatal death 9.5%. Our MNHR data show maternal and fetal sepsis rates of 1.4% and 11%, respectively, postpartum hemorrhage rates of 5.8%, and a maternal death ratio of 246/100,000 deliveries. Since our study is population based and the others were not, a direct comparison between these studies is not possible, but the direction of the findings is similar.

The strengths of this study include its large sample size, varied community-based sites on 3 continents, data collected prospectively, pre-specified composite outcome that combined prolonged labor, obstructed labor, and failure to progress used at all sites. A registry administrator who often interviewed the mother and/or her family and the delivery attendant, which could have been a traditional birth attendant, nurse, nurse midwife, or physician, collected the data. The registry administrator also reviewed the medical record for additional data, if available. Differentiating between OL, PL, and FTP at the sites would have been difficult if not impossible given the clinical and diagnostic limitations of these settings. The complexity of isolating OL/PL/FTP clinically as the cause of any individual maternal, fetal, or neonatal death makes data collection and analysis difficult, so this analysis, which reports data from 7 sites, is intended to be descriptive and not definitive, in terms of the actual prevalence of OL/PL/FTP and outcomes related to this condition. For example, why Zambia experienced a lower rate and Pakistan experienced a higher rate of OL/PL/FTP relative to the other sites may reflect the true rate of OL/PL/FTP in those geographic regions, or, perhaps more likely, reflects some difference in how OL/PL/FTP was clinically defined and recorded in the field.

A few other findings were notable with respect this analysis. First, while overall maternal, fetal, and neonatal outcomes were significantly worse in the setting of OL/PL/FTP, the experience was compounded up to fourfold in the African sites. Whether these increased risks in the setting of obstructed labor reflect an access to care issue versus some pathophysiologic or clinical etiology is not clear from this analysis, but warrants further investigation given the significantly increased burden of morbidity and mortality observed with OL/PL/FTP in the African sites. Pakistan also had a notably higher rate of OL/PL/POL, which we believe reflects poorer quality maternal and child healthcare in that setting as compared to other registry sites [12].

The second interesting finding is that this analysis is at odds with other previously published papers regarding the demographics of women experiencing OL with respect to education. Previous analyses report that a risk factor for women experiencing OL is poor educational status, but in this study, the opposite was seen. Given that this finding could reflect confounding factors, a regression analysis including adjustment for maternal demographics, which did not change the direction of the original analysis. The explanation for this result remains unclear.

The final notable finding of this analysis, is that women delivering preterm had a reduction in OL/PL/FTP of about 10%. Gestational age is difficult to define accurately in these settings since many women do not know the dates of their last menstrual period, and few had a dating ultrasound. Acknowledging that the MNHR gestational age data are imprecise, we nevertheless found a trend toward significance suggesting that women with preterm deliveries are less likely to experience OL/PL/FTP.

OL/PL/FTP puts maternal, fetal, and neonatal lives at significant risk for a wide variety of adverse outcomes. This analysis suggests that vaginal delivery exacerbates all of the maternal, fetal, and neonatal outcomes evaluated in the setting of OL/PL/FTP while cesarean section appears to reduce these adverse outcomes, although not as much as might be expected. This is likely attributable to delays in diagnosis, at which point delivery by cesarean section may be too late to impact outcomes from a prolonged dysfunctional labor. In terms of the results regarding attendant at delivery and delivery location, it appears that many women with OL/PL/FTP are eventually arriving at appropriate delivery settings and being delivered by skilled attendants. However, it is likely that women with OL/PL/FTP are arriving in these settings too late to affect the primary outcomes. The overall conclusion of this analysis is that labor should take place in the presence of an experienced provider at the outset who can recognize the signs of OL/PL/FTP and determine whether or not further intervention is necessary to prevent the excess maternal, fetal, and neonatal morbidity and mortality that occurs in untreated cases.

## List of abbreviations used

OL: obstructed labor; CS: cesarean section; CPD: cephalopelvic disproportion; NICHD: *Eunice Kennedy Shriver* National Institute of Child Health and Human Development; MNHR: Maternal and Newborn Health Registry; OL/PL/FTP: obstructed/prolonged labor/failure to progress; HW: healthcare worker; TBA: traditional birth attendant.

## Competing interests

Data and presentation of information has not been influenced by the personal or financial relationship of the authors with other people or organizations. Authors have no financial or otherwise competing interests to disclose.

## Author’s contributions

MH conceived of the study, and participated in its design and coordination and drafted the manuscript. RLG participated in its design and edited the manuscript. OP, SS, FA, EC, WAC, AG, NFK, KMH, SSG, BK, RJD, AP, PLH, FE, EAL, MKT, DDW, EMM and RLG designed and monitor the MNHR study quality. SA, SSG, OP, SS, FA, AG, AP, MB, AM, AM, and FE oversaw field activities and quality monitoring. Data analysis was conducted by JM, DDW, EMM with input from RLG. All authors read and approved the final manuscript.

## Peer review

Reviewer reports for this article can be found in Additional file 1.

## Supplementary Material

Additional file 1 Click here for file

## Declarations

This article has been published as part of *Reproductive Health* Volume 12 Supplement 2, 2015: Research reports from the NICHD Global Network for Women’s and Children’s Health Research Maternal and Newborn Health Registry. The full contents of the supplement are available online at http://www.reproductive-health-journal.com/supplements/12/S2. Publication of this supplement was supported by grants from the Eunice Kennedy Shriver National Institute of Child Health and Human Development to RTI International.
