# Supplementary material for: A prospective population-based study of maternal, fetal, and neonatal outcomes in the setting of prolonged labor, obstructed labor and failure to progress in low- and middle-income countries
Source: Reprod Health. 2015 Jun 8;12(Suppl 2):S9. doi: 10.1186/1742-4755-12-S2-S9 (PMC4464213; doi:10.1186/1742-4755-12-S2-S9)
Supplement: Additional file 1 [file 1742-4755-12-S2-S9-S1.pdf]

Referee's comments to the authors– this sheet WILL be seen by the author(s) and published with the article

Title	A prospective study of maternal, fetal, and neonatal outcomes in the setting of prolonged labor, obstructed labor and failure to progress in low- and middle-income countries
Author(s)	Margo S Harrison, Sumera Ali, Omrana Pasha, Sarah Saleem, Fernando Althabe, Mabel Berrueta, Agustina Mazzoni, Elwyn Chomba, Waldemar A Carlo, Ana Garces, Nancy F Krebs, K Michael Hambidge, Shivaprasad S Goudar, SM Dhaded, Bhala Kodkany, Richard J Derman, Archana Patel, Patricia L Hibberd, Fabian Esamai, Edward A Liechty, Janet L Moore, Marion Koso-Thomas, Elizabeth M McClure and Robert L Goldenberg
Referee's name	Michael Gravett

When assessing the work, please consider the following points, where applicable:

1. Is the question posed by the authors new and well defined?
2. Are the methods appropriate and well described, and are sufficient details provided to replicate the work?
3. Are the data sound and well controlled?
4. Does the manuscript adhere to the relevant standards for reporting and data deposition?
5. Are the discussion and conclusions well balanced and adequately supported by the data?
6. Do the title and abstract accurately convey what has been found?
7. Is the writing acceptable?

Please make your report as constructive and detailed as possible in your comments so that authors have the opportunity to overcome any serious deficiencies that you find and please also divide your comments into the following categories:

- Major Compulsory Revisions (which the author must respond to before a decision on publication can be reached)
- Minor Essential Revisions (such as missing labels on figures, or the wrong use of a term, which the author can be trusted to correct)
- Discretionary Revisions (which are recommendations for improvement but which the author can choose to ignore)

Where possible please supply references to substantiate your comments.

When referring to the manuscript please provide specific page and paragraph citations where appropriate.

General comments:
Maternal and neonatal morbidity and mortality remain important global health problems. Target goals to reduce maternal and childhood mortality, as outlined in the Millennium Development Goals 4 and 5, will not be met. Obstructed or prolonged labor remain important causes of both maternal and neonatal mortality and morbidity.

The great strengths of this manuscript are that it is one of the few prospective studies of obstructed labor and adverse maternal and fetal/neonatal outcomes that used standardized definitions, was multinational, had a large sample sizes and nearly complete (99.8%) reporting. Additionally, it is well written, with a thoughtful discussion.

While the findings are not necessarily new or unique, they validate previous smaller studies and clearly affirm the relationships among obstructed or prolonged labor and adverse outcomes. It is, therefore, an important contribution to the literature that will stimulate much interest in the realm of maternal child health.

A few discretionary revisions are suggested, as outlined below, that may strengthen the manuscript.

Major compulsory revisions:
None

Minor essential revisions:
None

Discretionary revisions:
Page 4. The population is well characterized, but it would be useful to know the distribution of deliveries by birth attendant and location, e.g., skilled birth attendant, traditional birth attendant, home or facility delivery. This may help explain:

- Disparities in OL/PL/FTP between sites since ascertainment bias between providers may affect reported rates, as noted on page 5, para 3.

Page 6. Delivery by Cesarean did not impact many of the primary outcomes. Could this have been attributed to delays in diagnosis-to-delivery, based upon site of delivery, type of birth attendant, and availability of emergency OB care?
Figure 1. Could the authors comment more specifically in the wide disparities in OL/PL/FTP rates between Pakistan and other countries? Might this be attributable to better access to health care facilities in Pakistan or maternal characteristics like earlier age at conception, etc?

Finally, one of the most significant sequela of obstructed labor is obstetrical fistula, particularly in Africa. Do the authors have data on the rates of obstetrical fistula following OL/PL/FTP? This would be especially relevant, since most reports of obstetrical fistula are restricted to patients presenting to a facility for repair. The large prospective cohort presented here would be very helpful in ascertaining population based rates of obstetrical fistula associated with obstructed labor. This may be the basis for an additional manuscript.

Supplement Editor's comments
One important asset of you analyses is that information is coming from communities and is population based. Please make clear this concept all over the text including the title and abstract.
In references not coming from Journals or books please provide the link and date accessed.
